# Pancreatic tumor initiation: the potential role of IL-33

**DOI:** 10.1038/s41392-021-00636-x

**Published:** 2021-05-22

**Authors:** Xing Huang, Gang Zhang, Tingbo Liang

**Affiliations:** 1 Department of Hepatobiliary and Pancreatic Surgery, the First Affiliated Hospital, Zhejiang University School of Medicine, Hangzhou, Zhejiang China; 2 Zhejiang Provincial Key Laboratory of Pancreatic Disease, the First Affiliated Hospital, Zhejiang University School of Medicine, Hangzhou, Zhejiang China

**Keywords:** Gastrointestinal cancer, Cancer microenvironment

A recent report published in *Nature* by Alonso-Curbelo et al.^1^ revealed epigenetic reprogramming in pancreatic cancer initiation caused by the cooperative effects of *KRAS* mutation and environmental damage. In the report, interleukin-33 (IL-33) was further identified as the effector that dominates environmental damage-induced early neoplasia and neoplastic transformation in the presence of *KRAS* mutation. This study thereby provides a specific molecular mechanism to understand the gene–environment interaction with regard to the processing of pancreatic cancer initiation.

Today, there is a consensus that intrinsic elements (e.g., gene mutation and alteration) and extrinsic factors (e.g., environmental stimulation and stress) co-contribute to tumor initiation. In pancreatic cancer, the critical role of internal gene mutations in tumorigenesis is well-documented. For instance, *KRAS* is identified as the most frequently mutated oncogene in more than 90% of pancreatic cancer patients, and the constitutively active *KRAS*^G12D^ mutation has been proved to facilitate the formation of pancreatic intraepithelial neoplasia. However, *KRAS* mutation alone is characterized by weak oncogenicity that is not enough to drive pancreatic tumorigenesis. Thus, it has long before been recognized that environmental factors must be indispensably involved in the initiation of pancreatic cancer. Environmental disturbance usually triggers a process termed as acinar-to-ductal metaplasia (ADM). Tissue damage together with *KRAS* mutation causes anomalous persistence of ADM, subsequent progression into pancreatic intraepithelial neoplasia, and eventual pancreatic ductal carcinoma (PDAC). Unfortunately, given the various complexities of microenvironmental influences on pancreatic cancer,^2^ the underlying molecular mechanisms by which the external environment stimulates pancreatic tumorigenesis are still not fully understood. Recently, Alonso-Curbelo et al. revealed that in combination with *KRAS* mutation, environmental damage unleashes the epigenetic remodeling program for early neoplasia and neoplastic transformation.

As the first step toward understanding how genetic elements and environmental factors interplay to reprogram the pancreatic epithelium during PDAC development, Alonso-Curbelo et al. mapped chromatin accessibility of pancreatic epithelial cells across normal, injured, *KRAS*-mutated, *KRAS*-mutated plus injured, and malignant tissues using genetically engineered mouse models (GEMMs). Within 2 days after injury-mediated tissue damage, cells with *KRAS* mutation underwent synchronous neoplastic reprogramming and showed a chromatin accessibility profile analogous to that of PDAC cells. Of note, most open chromatin regions were marked to noncoding intergenic and intronic regions binding to master transcription factors that control pancreatic cell lineage commitment and carcinogenesis. Further evaluation showed that tissue damage and *KRAS* mutation synergically promote chromatin accessibility of the enhancers involved in advanced PDAC. To functionally associate these chromatin changes to cell-fate transitions in vivo, the authors incorporated pancreas-specific shRNA to perturb the production of *BRD4*, an epigenetic regulator and reader that recruits regulatory complexes to acetylated chromatin and is particularly important for enhancer-mediated transcription of cell-identity genes. The results revealed that *BRD4* deficiency inhibited injury-induced ADM progression to pancreatic intraepithelial neoplasia in the presence of *KRAS* mutation. The authors further found *BRD4* inhibition blunts the expression of genes associated with acinar morphology, and suppresses the *KRAS* effectors, cancer-associated transcriptional targets, as well as advanced PDAC-characteristic genes. Therefore, after injury-induced chromatin changes, the *BRD4*-dependent transcriptional reprogramming is a key downstream event in pancreatic cancer initiation. While identifying potential mechanisms by which the injury-induced ADM progresses to pancreatic intraepithelial neoplasia, the authors observed a *BRD4*-dependent gene-regulatory program uniquely induced by the combined effects of mutated *KRAS* and tissue damage, which was largely similar to that observed in advanced disease and was strongly associated with characteristic signatures defining human PDAC. By performing single-cell ATAC-seq on over 6000 *KRAS*-mutated cells isolated from *KRAS*-mutated or *KRAS*-mutated plus injured conditions, the neoplasia-specific chromatin state was confirmed as bona fide chromatin remodeling. Thus, tissue injury and oncogenic mutations cooperatively remodel chromatin, thereby producing neoplasia-specific transcriptional programs. In search of chromatin-activated neoplasia effectors, the authors observed a rapid increase in alarmin cytokine IL-33 as well as a swift and selective gain of IL-33 locus in *KRAS*-mutated epithelial cells undergoing injury-facilitated chromatin switch in a *BRD4*-dependent manner. Moreover, exogenous IL-33 mimicked the effects observed in case of injury and cooperated with *KRAS* mutation to activate the neoplasia-specific and *BRD4*-dependent gene expression program, associated with an accelerated appearance of pancreatic intraepithelial neoplasia. Thus, IL-33 acts as a chromatin-activated effector in neoplasia development of pancreatic cancer. Collectively, this study revealed that environmental damage, in combination with *KRAS* mutation, induces *BRD4*-dependent epigenetic reprogramming, as well as identified IL-33 as an effector of environmental damage to drive early-stage neoplasia for pancreatic cancer initiation.

The construction of preclinical models plays an essential role in the exploration of PDAC progression and treatment.^3^ The existing models are mainly classified into cell lines, organoid and patient-derived xenograft (PDX), as well as GEMMs. The first three forms have several weaknesses, necessitating the use of GEMMs in preclinical trials. For example, 2D co-culture is inconsistent between passages and unable to completely model tumor heterogeneity, intercellular contact, structure, or gene expression. Further, PDX models fail to represent the induction of immune privileges in the process of tumor development and their original stroma is usually replaced by host components, leading to genetic and functional drifts away from the primary tumors. In contrast, GEMMs are characterized by endogenous stromal and extensively integrated immunosuppressive mechanisms, and thus, have become the most widely applied model to decrypt and manipulate the regulatory mechanisms of cancer initiation and progression. Concomitant mutation of *KRAS* and *TP53* is currently the main approach to spontaneously induce pancreatic cancer initiation. However, such a conventional strategy is highly time-consuming and usually requires more than six months. According to the findings of Alonso-Curbelo et al., the initiation of PDAC can be largely promoted by environmental insults. In fact, the neoplasia-specific epigenetic reprogramming was observed within two days after injury in *KRAS*-mutated pancreatic epithelial cells. More importantly, as a critical effector in this program, IL-33 can replace the need for injury to accelerate the formation of early neoplastic lesions, simultaneously avoiding potential injury-caused side or artificial effects. Therefore, IL-33 is a promising booster for spontaneous PDAC GEMMs, potently shortening the construction cycle.

**Fig. 1:**
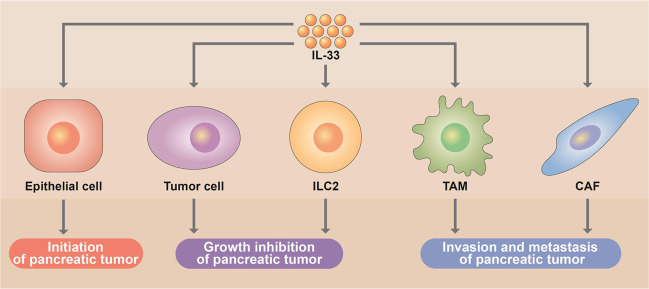
Functional complexity of IL-33 in pancreatic cancer. The schematic model highlights that the epithelial cell, tumor cell, ILC2, TAM, and CAF are individually or jointly involved in IL-33-mediated initiation, growth inhibition, invasion, and metastasis of pancreatic tumor, indicating the multi-faceted roles of IL-33 in tumorigenesis and progression of pancreatic cancer

Actually, IL-33 is a member of the IL-1 family that plays key roles in both innate and adaptive immunity by stimulating multiple immune cells. It is constitutively expressed by an abundance of endothelial, epithelial, and fibroblast-like cells. In the state of injury, necrosis, or cell stress, IL-33 is released into the extracellular space and binds to its cognate receptor interleukin-1 receptor-like 1 (IL1RL1), which has been associated with tissue and metabolic homeostasis, inflammation, infection, and cancer. Given the widespread expression and multiple downstream functions of IL-33 as well as the difficulty in identifying pancreatic cancer initiation clinically, IL-33 may not be a direct target for the prevention or early diagnosis of pancreatic cancer. Instead, further exploration of detailed molecular mechanisms by which IL-33 induces pancreatic cancer initiation may be favorable for defining novel targets to prevent its onset. Interestingly, in addition to pancreatic cancer initiation, accumulating evidence suggests differential effects of IL-33 on the development and progression of PDAC (Fig. 1). For instance, Moral et al.^4^ revealed that IL-33 restricts PDAC growth by activating group II innate lymphoid cells (ILC2s), which recruit CD103^+^ dendritic cells, triggering the recruitment and priming of T cells. In contrast, Andersson et al.^5^ reported that cancer-associated fibroblast (CAF)-derived IL-33 induces the transition of tumor-associated macrophages (TAMs) to the M2 phenotype and mediates CAF-TAM-committed PDAC metastasis via the IL-33-NFκB-MMP9-laminin axis. Together, IL-33 plays complex roles in pancreatic cancer, and therefore, deserves to be further investigated for future targeting.

Given many human body tissues (e.g., blood, skin, and gut epithelial cells) are often subjected to environmentally mediated damage, the study by Alonso-Curbelo et al. provides a basis for investigating gene–environment interaction-induced cancer initiation in other tumor types. Nevertheless, their findings are still not be confirmed in human pancreatic cancer, and the clinical significance of their findings requires in-depth exploration.
